# Congenital Occlusion of the Posterior Nares*Read before the New York State Medical Association, in New York, Nov. 17, 1886.

**Published:** 1886-12

**Authors:** Alvin A. Hubbell

**Affiliations:** Buffalo, N. Y.; Professor of Diseases of the Eye, Ear, and Throat in the Medical Department of Niagara University; 212 Franklin street


THE BUFFALO MEDICAL AND SURGICAL JOURNAL

VOL. XXVI. DECEMBER, 1886. No. 5

### Original Communications.

### *CONGENITAL OCCLUSION OF THE POSTERIOR NARES.**

BY ALVIN A. HUBBELL, M. D., BUFFALO, N. Y.

Professor of Diseases of the Eye, Ear, and Throat in the Medical Department of Niagara University.

Because of the rare occurrence of congenital occlusion of the posterior nares (choanæ), I have been constrained to place upon record a case of this interesting deformity which I have had the opportunity of observing and treating, and also to accompany the report with brief notes of such other cases as I have been able to find in surgical literature.

On September 1st, 1883, G. R. M., a young man, eighteen years of age, was brought to me by Dr. L. W. Tarbox, of South Dayton, N. Y. He gave me the following history: He was the tenth child of a family of eleven children, all of the rest of whom were well-formed and generally free from any physical defects, excepting that two sisters had obstruction of one nostril from "vomer bone growing to one side." Soon after birth, it was noticed that he could not breathe through his nose. It was impossible for him to suckle, and, while very young, much trou-

*Read before the New York State Medical Association, in New York, Nov. 17, 1886.

ble was experienced in keeping his mouth open so that he could breathe through it, especially when asleep. He was a vigorous baby, and, notwithstanding this drawback in respiration, he thrived well and grew rapidly. As he became older, his sleep was less disturbed by difficult breathing, the greatest annoyance then experienced arising from the lips, mouth, and throat becoming very dry. He had never had any sickness, excepting an attack of croup when he was three years old.

At this consultation he was a strong, well-developed young man, somewhat short in stature, and weighing about 160 pounds. He breathed entirely through the mouth, being obliged to keep it more or less open for this purpose, thus giving him an unpleasant facial expression. His words were spoken with an entire absence of the nasal sounds. He said it was "harder for me (him) than others to drink, swallow, or chew food." Taste was good. The sense of smell was "very weak," if he could "smell at all." Mucus discharged freely from the nose, and was a source of much annoyance to him. His hearing was acute, and he has never had disturbances of any kind in his ears. The membranæ tympani were normal in appearance, and the Eustachian tubes were freely permeable, permitting easy inflation of the tympanic cavity. The nose was quite large and its cavities, anteriorly, were of normal shape and full size. The mucous membrane, however, lining these cavities was more or less swollen and congested. The inferior meatus on either side was large, and extended backward to a depth of five centimetres (one and a half inches), when an obstruction was met which was firm and complete. An examination of the posterior nares, with the index finger passed behind and above the palate, showed a well-marked, somewhat conical depression on each side, about six millimetres (one-fourth of an inch) deep. The posterior border of the vomer was prominent, and distinctly separated the two depressions. A rhinoscopic examination was not practicable.

To determine the character of the obstructing partition and the line of treatment to be pursued, I passed a medium-sized trocar through the one on the left side. I found the occlusion to be a thin, bony plate, covered on both sides with mucous membrane. It was penetrated without much force by giving the instrument a drilling or rotatory movement. Having thus determined the character of the obstruction, it was deemed best to make as large an opening as possible by means of a drill as large as the inferior meatus in front would admit.

The patient was admitted to the Buffalo Hospital of the Sisters of Charity, and the operation was made Sept. 21st, with the assistance of Dr. Tarbox and residents Murphy and Hill. The patient was anæsthetized with chloroform, and with a hand-drill having a diameter of thirteen millimetres (over one-half inch), and with edges well sharpened, each partition wall was carefully penetrated by rotatory movements of the instrument, and free openings made. There was considerable hemorrhage, but this soon ceased. Breathing through both sides of the nose took place at once, and was free and easy. After recovery from the anæsthetic, the patient expressed himself as feeling a sense of comfort which he had never known before. He was kept in bed a day or two, and the nose was cleansed with carbolized water. To keep the openings sufficiently large and free, sounds and tents were afterwards introduced regularly twice a day. The patient was discharged September 29th, with nasal respiration easy, both when awake and asleep.

November 17th, the patient returned to me with the openings so much contracted, notwithstanding the efforts to keep them large, that breathing through them was somewhat difficult, and it was necessary for him, during a part of the time, to breathe through the mouth. I decided to repeat the previous operation and endeavor to keep the openings of sufficient capacity by inserting as large-sized tubes as possible, and retaining them till the healing process was nearly or quite complete. I had tubes of "block tin" made of two sizes from which to choose, the diameter of one size being ten millimetres (less than one-halt inch), that of the other thirteen millimetres (more than one-half inch), and their length five centimetres (two inches). The patient having been placed in the hospital as before, he was again anæsthetized with chloroform, when I proceeded to repeat the previous operation with the drill, my colleague, Dr. John Cronyn, and Drs. Tarbox, Hill and Murphy kindly assisting. After the hemorrhage from the operation had ceased, the parts were cleansed with carbolized water, and an attempt was made to introduce the larger tubes. These were found to be too large to be safely used, on account of their making severe pressure on the vomer, alæ, inferior turbinated bones, and surrounding parts. The smaller tubes were, therefore, introduced, and proved to be of ample size for the parts without making undue pressure at any point. When in place, they were almost entirely hidden from view, and posteriorly they projected to a plane corresponding with that of the posterior margin of the vomer. Previous to their introduction, a small cord was inserted into a hole in the anterior extremity of each tube to facilitate removal. This, when not in use, was placed within the tube out of sight. The patient breathed easily through the tubes, and he was permitted to leave the city November 24th, with directions to cleanse the parts as effectually as possible, and as frequently as seemed necessary, with carbolized water.

On December 28th, about six weeks after the operation, the tube in the right nostril was accidentally withdrawn by the patient, and on January 5th, 1884, about seven weeks after the operation, I removed the one in the left side. Both tubes were held in place quite firmly at first, but afterward were loosened, and came away easily. After removal of the tubes, the openings through the obstructing partition were found almost entirely healed, and they were fully as large as the diameter of the tubes. The case has been seen recently, and, although the openings have contracted some, the space is sufficient for free nasal respiration.

After the operation, the patient could not at first properly regulate the action of the palate in speech, so as to cut off certain sounds that should not pass through the nose, and his articulation was, therefore, too nasal. In a short time, however, he so educated the palatal muscles as to be able to give the sounds of the vowels and of such consonants as g, k, b, etc. The acts of mastication and swallowing were accomplished with ease, the mouth was kept closed as in other persons, and the facial expression was changed so as to appear perfectly normal. The sense of smell had not improved, but the anosmia continued complete, indicating, undoubtedly, a want of development of the olfactory nerve.

A point worthy of emphasis is the fact that there had never been any disturbance of hearing, and the Eustachian tubes, tympanic cavities, and membranæ tympani were normal in all respects, although the conditions of Toynbee's experiment were constantly present. As is well known, this experiment consists in tightly closing the nostrils, when continued acts of swallowing will exhaust the air within the tympanic cavities to the extent of materially disturbing the equilibrium of atmospheric pressure between the middle and external ear, and thus inducing changes, observable by the surgeon, and felt, even to a distressing degree, by the patient.

A careful examination of medical literature shows but few reported cases of congenital stenosis of the choanæ. The first reference that I can find made to this condition was by Adolph Wilhelm Otto, M. D.,* of Breslau, in 1831, who said: "In congenital closure of the hinder opening of the nostrils, the palatine bones are very much deformed." Otto thus implies that such cases had been seen, but he does not cite any.

Karl Emmert† was the first to record a case which came under his observation in 1851. The patient was a boy seven years old who had been unable to breathe through the nose from birth. He had been nourished with great difficulty during infancy, and often had suffocative attacks during sleep. Both choanæ were entirely closed, and mucus and tears were constantly discharging from the nose, and the sense of smell was wanting. The occlusion was found, both by preliminary examination and by operation, to be bony, but it was impossible to ascertain in what manner, or from which bones the partition wall arose.

* Compendium of Human and Comparative Pathological Anatomy, Translated by John F. South, London, 1831, p. 181.

† Lehrbuch der Chirurgie, Stuttgart, 1853, Bd. II., S. 355.

Emmert penetrated the obstructions with a specially constructed trocar, and enlarged the openings by the introduction of a catheter. Tubes were also introduced, and worn at times for half a year. The father of this boy had had syphilitic disease of the pharynx.

The next record was made by Luschka,* in 1859. He describes the case of a female infant who died shortly after birth, in whom both posterior nares were occluded, and in whom, also, many other deformities existed. A post-mortem examination showed that the occlusion on each side was a bony plate which extended from the palate bone, upwards and backwards, to the lower surface of the sphenoid. Laterally, each plate reached to the inner side of the internal plate of the pterygoid process, while in the median line they approached each other, being separated by a narrow slit which received the posterior extremity of the rudimentary vomer, and were united below where the posterior nasal spine usually arises from the palate bones.

R. Voltolini,† in 1871, gives the history of a medical student who suffered with closure of the right choana, which was apparently membranous, and due to "congenital adhesions." It was successfully opened by means of the galvano-cautery.

Fraenkel‡ mentions a case sent to him by Dr. J. Wolff, in which there was a bony closure of the posterior nares on the right side. The patient was a young man "who had been unable to blow his right nostril," and "had been excessively troubled by the accumulations sometimes escaping in front." Wolff had established the diagnosis by means of palpation of the pharynx and probing the nasal canal, and had perforated the bony partition-wall by "an operation." Fraenkel satisfied himself that the obstruction was a "smooth and solid wall, covered on both sides with mucous membrane, and closing the right fossa in precisely the same manner described by Luschka. The crista of the septum showed itself, even on the closed side, as a narrow strip.

* Arch. f. Path. Anat., XVIII., 1859; also der Schlundkopf des Menschen, Tübingen, 1868, S. 27.

† Die Anwendung des Galvanokaustik im Innern des Kehlkopfes und Schlundkopfes, Wein, II. Aufl., 1871, S. 260.

‡ Ziemssen's Cyclopœdia of the Practice of Medicine, 1876, Vol. IV, p. 113.

No other abnormality could be seen." The opening which Wolff had made contracted to the size of a pea, but afforded entire relief.

Bitot* (misprinted "Betts" in Mackenzie, *On the Nose and Throat*), of France, relates a case of "atresia or obstruction of the posterior orifices of the nasal fossæ, by two triangular bones (naso-palatine bones)," of which the *New York Medical Journal* makes the following note: In a fœtus of seven months the nasal fossæ "posteriorly were imperforate, the obstruction being due to the presence of two triangular bones of a more or less regular shape, articulating above with the sphenoid, below with the *os quadratum*, or the horizontal portion of the palatine bone. Exteriorly their borders correspond with those of the internal wings of the pterygoid apophyses, while internally they infringed one upon the other and formed a median fissure."

E. Zaufal,† of Prague, published, in 1876, the description of the case of a girl, fifteen years of age, with congenital stenosis of the right choana. The obstruction was bony, and was a little in front of the posterior edge of the choana, and at a distance of five and a half centimeters from the front. The plate was placed across the aperture in an oblique direction, extending from within, outwards and backwards, and from below, upwards and backwards. On the left side the nasal cavities were normal, and on the right, in front of the obstruction, of full size, but the mucous membrane here was swollen and yielded a free secretion, and the edge of the nostril was excoriated. The forehead was high and straight, and the eyes were slightly prominent. There was blepharitis, but the patient's vision was good. The hearing was acute, and the voice natural, without any nasal character. The sense of smell was absent on the obstructed side, but acute on the other. No operation was performed.

The next case in chronological order was recorded by Dr. J. Solis Cohen,‡ of Philadelphia, in 1879. It was that of "an

* Archiv de Toxocologie, Sept., 1876, (N Y. Med. Jour., July, 1877, pp. 92 and 97.)

† Prager Medicinische Wochenschrift, 1876, No. 45, p. 837.

‡ Diseases of the Throat and Nasal Passages, Second Ed., 1879, p. 385.

infant" who had great difficulty in suckling and breathing and suffered frequent suffocative paroxysms. The child was relieved by "boring through the occluding structures with a knife and steel probe," which was followed by the insertion of a sound, from time to time, and the introduction of small bits of sponge securely fastened to a holder.

Dr. T. G. Morton,* of Philadelphia, treated the following case in the Pennsylvania Hospital: "John B. æt. eight years, was admitted for almost an entire occlusion of the left side of the mostril. The affection had existed from infancy. Had been treated on several occasions without benefit. On being brought to the hospital, Dr. Morton found that a delicate probe could be pushed back between the sides of the nose at its lower part. A sponge tent was then introduced, which, after remaining for four days had stretched the parts sufficiently for the passage of a large catheter. The opening showed no disposition to close. The child was subsequently brought to the hospital for examination, and the result was entirely satisfactory."

Dr. T. R. Ronaldson,† of Edinburgh, gives the notes of a case which he saw in 1881, of a plump and well developed female child, which was delivered after a short and easy labor, in whom existed obstructions of the nasal passages. When it was born it was noticed that the breathing was not natural, and in attempts to inspire "the under lip and cheeks were sucked in," and the lungs were not inflated. It was made to cry by slapping the buttocks, when respiration took place, which was afterwards free, when the mouth was open and the tongue slightly pulled forward. The nostrils were examined and found filled with a translucent, glue-like substance, which was removed *en masse*. Attempts were made to blow air through the nares, and to pass a probe, but without success.

The case was diagnosed as an "organic obstruction of the posterior nares." Whenever a point of moderate asphyxia was reached, the child would open its mouth to cry, and would thus inflate its lung. Hoping that respiration would be thus kept

* Surgery in the Pennsylvania Hospital, 1880, p. 333.

† Edinburgh Medical Journal, May, 1881, p. 1035.

up, the case was left with the intention of doing something for its relief later. But contrary to the hopes of the doctor, the child died an hour after his departure.

An imperfect post-mortem examination showed that the posterior nares were completely occluded by a firm membrane, through which an ordinary surgical probe "could hardly be forced without bending on itself." No other abnormality was found, "the nose, anterior nares, the hard and soft palate being normal."

Dr. Oren D. Pomeroy,* of New York, reported a case, in 1881, in which there was bony occlusion of the right nostril in a man forty-four years of age, which had existed "longer than he could remember." There was abundant catarrhal secretion, which was rather offensive. The closure of the nostril was a source of embarrassment in cleansing it. The nares, both anterior and posterior, were narrow. Dr. Pomeroy states that the obstruction was about half an inch anterior to the extremity of the posterior nares, that it was a "solid wall of bone" and "of no great thickness." There was considerable deafness on the affected side. He perforated the bone by drills, and using, finally, "a cross-cut burr drill," the head having an almond shape, and as large as could be conveniently introduced into the nostril—about two and a half lines in diameter. After three operations the opening was sufficiently large "for moderately difficult respiration through the nostril."

At a meeting of the New York Society of German Physicians, held May 27th, 1881, Dr. Richard C. Brandeis,† of New York, showed a young girl who had come to him with a nasal obstruction of the left side. After removal of several polypi, he found a complete bony occlusion about one and three fourths inches from the nostril. The bony plate was perforated by means of the galvano-cautery, and nasal breathing was entirely restored.

* Transactions of the N. Y State Medical Society, 1881, p. 200; also Pomeroy's Diseases of the Ear, 1883, p. 201.

† New York Medical Record, Nov. 12, 1881, p. 552.

Dr. T. B. Wilkerson,* of Young's Cross Roads, N. C., related, in 1882, the following case: W. C., æt. six years, has had no nasal respiration since birth; and when an infant was hand-fed, being unable to nurse from the mother's breast. The anterior outlets of the nasal passages were very small, "scarcely admitting the ordinary silver catheter," and the fossæ presented an irritated appearance. The edges of the eye lids were red, and there was occasional "flooding" of the eyes. The mouth was open. The general mental and physical condition of the patient was good. There was no deafness, but the "special senses of smell and taste were very deficient." Careful examination showed both nasal cavities closed at their posterior outlets. The obstructions were opened by a revolving trocar and canula, made especially for the case. "Gum-tubing" with holes cut in it at different points was inserted in each side and allowed to remain six days, the parts being cleansed, in the meantime, by syringing through the tubing. A gum bougie was afterwards passed every day. The channels remained pervious, and nasal respiration was established, "adding greatly to the comfort of the patient."

Dr. Sommer,† of Prague, presented to the medical society of that city, in 1883, a lad, nineteen years of age, in whom there was a congenital bony occlusion of the left choana. The head had somewhat the shape of a hydrocephalic. The external nasal framework was regularly developed and no abnormality was to be observed about the face, or in the cavity of the mouth. The right nasal cavity was wholly pervious, but the left was impenetrable, as neither air by expiration or inspiration, or water by injection, could be forced through it. The secretion of normal mucus was constantly discharging from its anterior opening, and the sense of smell was entirely wanting on this side. By anterior and posterior examination, the left choana was found entirely occluded by an obliquely standing bony plate, covered with mucous membrane. The patient declined to be operated upon.

E. Zaufal,‡ according to the reporter of Sommer's case above, mentions a second case which had come under his observation.

*N. C. Medical Journal, June, 1882, p. 305.

†Weiner Medizinische Presse, April, 1883, No. 15, p. 476.

‡Weiner Med. Presse, April, 1883.

A perfectly healthy student fourteen years old, had stenosis of the left side of the nose, which was "wholly identical" with that of Sommer's case.

Prof. L. v. Schrötter,* of Vienna, last year gave a detailed description of a case which he had treated the year before (1884). The patient M. Auer, æt. nineteen years, was a strong and well-developed farmer's daughter with occlusion of both choanæ which had existed from birth. The facial expression was very striking, being marked by slightly prominent eyes, mouth open, under lip somewhat hanging, naso-labial folds wanting, and the action of the alæ of the nose deficient. The secretion from the nose was free when lachrymation was increased, as in crying or long exposure to the cold air. The mouth and throat were most of the time dry, and there were occasional headaches. The hearing was below normal, taste was poor, and there was absolutely no sense of smell. The speech was a little difficult and nasal in character.

Prof. Grüber found the membranæ tympani deeply drawn inwards. The nasal fossæ were of normal size in front, but the mucous membrane, in the lower parts of these cavities, was "granulated, swollen, and congested." Posteriorly all parts were normal, except the mucous membrane above, which was swollen. Both choanæ were closed with diaphragms which were set a little in front of the posterior orifices. They were at first considered membraneous, but were, after operation, found to be bony. They were thickest near the outer parts, and their direction was from below, upwards and backwards.

The treatment consisted in the repeated application of the galvano-cautery and latterly the use of a covered chisel, to remove the thicker bony parts which the cautery did not readily destroy. The treatment was continued for several weeks. The openings were then of sufficient size for easy nasal respiration, and the patient was finally discharged May 13th, (1884). She was then able to breathe freely through the nose, the speech and facial expression had greatly improved, and the sense of smell was regained, although imperfectly.

* Monatsschrift für Orenheilkunde, Berlin, April, 1885, p. 97.

Dr. W. E. Casselberry,* of Chicago, relates, in 1885, the case of M. E., a Russian Polander, æt. about forty years, who had suffered during the past thirteen years from an obstruction of the nasal chambers. "Examination showed that a tense membrane covered the left choana almost completely." Its free edge was thin and sharp, and approached so near the septum narum that there was only a very small chink between the two. It could be pushed backward by the probe, and was from one to two millimetres in thickness. "The choana on the right side was partially covered by a membrane, which extended about half way across the aperture." This membrane was much thicker and less tense than that on the left side. The pharyngeal mouth of the Eustachian tube could not be seen on either side with the rhinoscope, the membranes evidently lying behind them. There were many distressing symptoms in this case from mouth-breathing and naso-pharyngeal catarrh. "Deafness in the left ear and annoying tinnitis aurium had long been prominent symptoms." There were also numerous head symptoms.

The obstructions were fully overcome by means of the galvano-cautery, the operation being repeated on each side three or four times. As soon as the nasal respiration was freely established, the patient was completely relieved of all cephalic symptoms, including the left-sided deafness.†

Another case has been cited by Dr. G. M. Lefferts,‡ of New York, and is credited by him to Gosselin. But I am unable to verify the doctor's reference; and as I cannot find the case anywhere, I do not include it my report.

I have now presented a brief review of sixteen cases, besides my own, using, so far as possible, the phraseology of the original reports. The total number of cases included in this paper is, therefore, seventeen. A brief analysis of these shows that ten were males, five females, two, sex not mentioned. Two died soon after birth, one was a seven months fœtus, and fourteen

* Journal of the American Medical Association, Aug. 8, 1885, p. 148.

† I include this among the list of cases of congenital occlusion, as the history, as given by a patient, is often very unreliable, and as I cannot conceive of an obstruction of this kind as originating otherwise than congenitally. Dr. Casselberry also suggests that "it is most likely" congenital.

‡ International Encyclopœdia of Surgery, 1885, Vol. V., p. 411.

were living at the time of the respective reports. Careful post-mortem examinations were made in two cases, an imperfect one in one case. The occlusion was complete in both posterior nares in eight cases, in the right in four, and in the left in three. It was incomplete in both nares in one case, and in the left naris in one. The occlusion was bony in twelve cases, and membranous in five. The two cases of incomplete occlusion were of the membranous variety.

As to the genesis of these obstructions, it appears that those which are bony are developed from the perpendicular or horizontal plate of the os palatinum, or both, and thence extend across the choanæ, usually in an oblique direction from below, upwards and backwards, and from within, outwards and backwards, to be united with the sphenoid above, and the vomer, or its opposite fellow at the median line. The connection of these plates with the palate bone is *indicated* by their situation in the living subject, and is *demonstrated* by the dissections that have been made.

Certain symptoms were found to be common to most of the cases. For instance, in bilateral occlusion there were the altered facial expression, difficulty in articulation, with nasal intonations, dryness of the mouth and throat, suffocative attacks and respiratory difficulties in infancy, inability to suckle, etc. In these cases, and in those with unilateral atresia as well, there were also an entire absence of the sense of smell on the affected sides, together with a constant mucous discharge, the discharge being increased whenever there was an extra secretion of tears. Usually the mucous membrane, corresponding to the side obstructed, was found swollen and congested, at least, anteriorly. Ear disturbances were sometimes noticed, and in my own case mastication and deglutition were also less easily performed than by other persons.

Regarding the treatment and results, I may add that the partition-walls have been opened in different ways, and the results have generally been satisfactory. The galvano-cautery, drills of different forms, trocars, chisels, knife, tents, tubes, etc., have all been effectually used. The galvano-cautery seems to have served the best purpose when the occlusion was membranous. Various reflex and distressing symptoms have been removed by opening the closed passages, and the senses of hearing, taste and smell have occasionally improved. In cases of double occlusion, immense relief has followed full and free nasal respiration, and with it a comfort to the patient never known before. The facial expression has become natural, and the articulation and intonation have much improved.

The prognosis is generally admitted to be unfavorable in infants, in all forms of stenosis of both nasal passages, from whatever cause. Some of the cases of double congenital occlusion have died soon after birth from undoubted asphyxia, and all of them have been reared with difficulty. This suggests timely relief by opening the obstructions without delay, in the meantime keeping the mouth open and tongue somewhat drawn forward, so that the child can breathe till nasal respiration is established by the various means that are always at the command of the practitioner.

212 Franklin street.

